# Out of the blue carbon box: toward investable blue natural capital

**DOI:** 10.1098/rsbl.2024.0648

**Published:** 2025-04-16

**Authors:** Catherine E. Lovelock, Carlos M. Duarte

**Affiliations:** ^1^ School of Environment, University of Queensland, St Lucia, Queensland, Australia; ^2^ Marine Science Program, King Abdullah University of Science and Technology, Thuwal, Makkah, Saudi Arabia

**Keywords:** coastal wetlands, conservation, restoration, carbon markets, nature-based solutions, nature finance

## Abstract

In 2019, we found that the concept of blue carbon had begun to solidify in the preceding decade around activities that could achieve mitigation through conservation and restoration and on ecosystems with high levels of data. Five years later, the available data have increased, and so too have the ecosystems that are included in national carbon markets and carbon market methodologies (e.g. seaweed and supratidal forests). While the implementation of blue carbon strategies continues to advance in both the carbon and emerging biodiversity markets, the scale of investment is inadequate for the action needed to meet global targets of the Paris Agreement and Kunming-Global Biodiversity Framework. The developing finance mechanisms for investment in blue natural capital offer additional potential for action on conservation and restoration of blue carbon ecosystems at large scales, although governance systems are challenged to deliver just and equitable outcomes. Blue carbon research and implementation is characterized by deep collaboration among diverse disciplines and actors, which continues to be crucial to achieving conservation and restoration goals.

## 1. Introduction—an expanding scope

The blue carbon concept, to mitigate climate change through the conservation and restoration of marine ecosystems, has led to a proliferation of projects delivering carbon benefits and saleable credits, dominated by mangrove restoration. The scope of blue carbon strategies has expanded beyond mangroves, tidal marshes and seagrass. Blue carbon encompasses the conservation, restoration and culture of kelp and other seaweeds, and proposals for management of tidal mud flats, continental shelf sediments, supratidal vegetation and other benthic ecosystems to maintain or build their carbon stocks and CO_2_ removal potential [1,2]. The expansion of the scope of blue carbon across multiple ecosystems is matched by an increasing scope in the motivation of the projects. The initial national and corporate policy motivation for blue carbon projects, meeting net zero climate mitigation goals and to a lesser extent adaptation and other co-benefits, is now being supplemented with the action required to meet the ambitious goals of the Kunming-Global Biodiversity Framework (KMGBF; https://www.cbd.int/gbf). Commitments to the KMGBF require, among many targets, to halt losses, protect 30% of land and ocean and restore 30% of degraded habitats, all by 2030, thereby aligning with the action required to deliver blue carbon projects [3]. Indeed, the KMGBF encourages nations to activate nature-based solutions, such as blue carbon projects, to the maximum possible ambition, because of the substantial benefits for biodiversity these conservation and restoration projects bring about.

Realizing the ambitious KMGBF goals for mangrove, seagrass and tidal marsh habitats will require an estimated USD$520 billion in funding by 2030, of which carbon credits may meet a substantial fraction, estimated at 40% [3]. Hence, carbon credits flowing from blue carbon projects need to be supplemented with additional financial resources to meet KMGBF targets for these ecosystems. To this end, the KMGBF further encourages governments to engage with the private sector to disclose the impacts, directly and through their supply chains, on biodiversity and encourage them to take action to contribute to the goals. As a result, there is an emerging focus on developing biodiversity certifications for the private sector (e.g. market-based methods). However, a narrow focus on markets for carbon and biodiversity credits, with limited consideration of other ecosystem services may lead to inconsistencies and delays in achieving blue carbon and KMGBF goals [4]. For example, a national focus on carbon may result in favouring mangrove over seagrass conservation, which at a local scale could limit conservation of biodiversity and fisheries ecosystem services. Conversely, a focus on biodiversity targets may result in limited investment in activities that enhance coastal protection, water quality or carbon sequestration. Thus, the implementation of additional approaches, including the accelerated adoption of blue natural capital as an investable asset class is needed [4].

While the earlier focus for blue carbon was on mangroves, seagrass and tidal marshes [5], here we reflect on the inclusion of additional coastal and marine ecosystems within the blue carbon concept. The increasing breadth of the blue carbon narrative to encompass biodiversity in these ecosystems broadens the opportunities to develop blue natural capital projects at landscape scales. This broader scope and, importantly, larger scale may lead to fast-tracking of blue carbon ecosystems as a new investable natural asset class. Therefore, we outline recent developments in finance for conservation and restoration of blue natural capital and evaluate their potential for accelerating conservation and restoration of blue carbon ecosystems. Finally, we suggest a research agenda that could stimulate investment in blue natural capital assets.

## 2. New science, expanding definitions of blue carbon ecosystems

In 2019, we evaluated blue carbon ecosystems against criteria that included the potential scale of CO_2_ emissions and removals, the permanence of carbon storage, the potential for management of ecosystems (and potential for negative impacts) and the alignment with international and national policies for mitigation and adaptation [5]. The science describing the carbon stocks and fluxes within these ecosystems and impacts of management, both on the ecosystems and socio-ecological systems in which they are embedded, has greatly expanded since that earlier assessment. Additionally, global policy imperatives include targets under the KMGBF [4,6]. In addition, the consideration of inorganic carbon dynamics [7] has led to an emerging body of evidence pointing at a significant component of CO_2_ removal through the alkalinity release from dissolution of carbonates in the rhizosphere of many blue carbon ecosystems [8,9].

Seaweeds, including kelp, were proposed to contribute to blue carbon stocks in the oceans since 2016 [10]. Since then, evidence suggests that approximately 15% of global seaweed production could be transported to the deep oceans and thus could be considered permanent with permanence defined as carbon stored for more than 100 years [11]. Evidence for avoided greenhouse gas emissions by using seaweed aquaculture products has increased and encompassed pathways like substitution for more climate-intensive products (e.g. the use of seaweed in feed for livestock and food products [12]). There is emerging evidence that carbon is accumulated in sediments below seaweed farms [13]. Indeed, carbon credits based on seaweed management are already being issued under Japan’s J-Blue carbon credit offsetting scheme [14], building on the culture of valuing and conserving seaweed in the country. Knowledge of the key biophysical and social factors that influence mitigation potential, feasibility and co-benefits is becoming established and informing actions for implementation of management of these ecosystems for genuine climate mitigation (e.g. [15]), and for the biodiversity and social co-benefits they offer [14].

**Figure 1.**
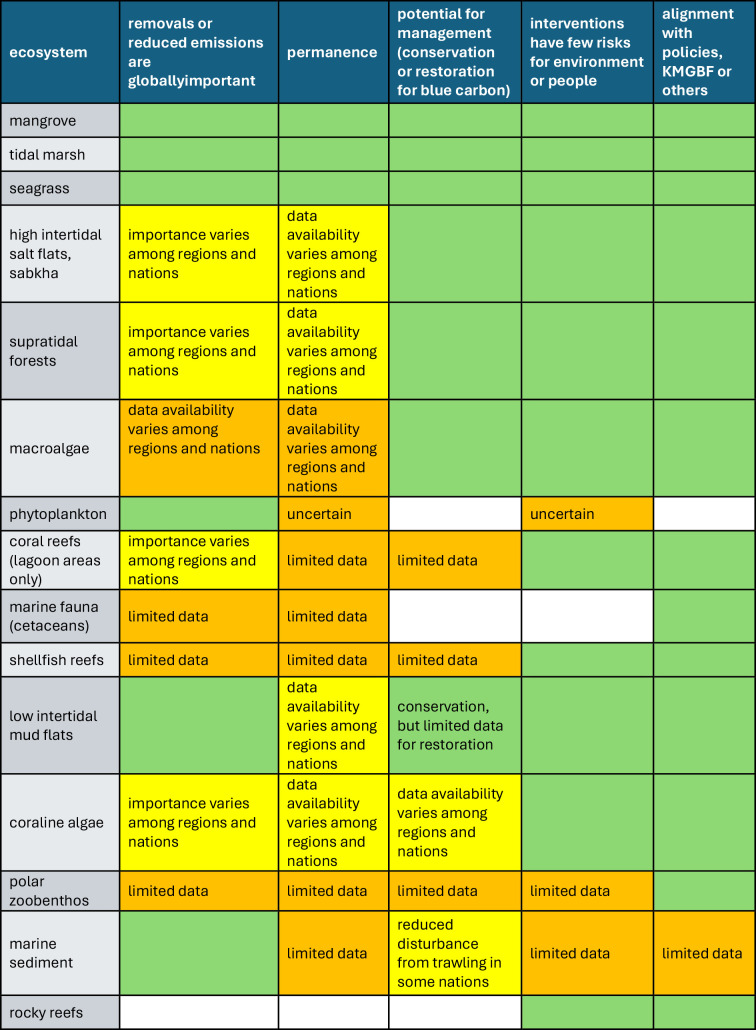
Evaluation of marine and coastal ecosystems against criteria (column headings) used to assess blue carbon ecosystems. Synthesis of evaluations from [1,2,5]. Green suggests ecosystems meet the criteria. White indicates they are unlikely to meet criteria or not applicable. Yellow indicates that evidence is available regionally or nationally. Orange indicates data availability is limited and/or confined to spatially limited case studies. The text in the boxes indicates limitations.

Other ecosystems that were in scope in 2019, or have come within scope since 2019, have differing levels of evidence available to confirm the scale and extent of carbon storage potential, the potential for management and/or the provision of co-benefits. As the science matured, knowledge of how these ecosystems meet the criteria, and the variability in their capacity to meet the criteria on local, national and regional scales has increased, supporting further research activity (figure 1). For example, supratidal forests, that are influenced by tides and groundwater, have high carbon stocks, are endangered on multiple continents and have high biodiversity and cultural significance [16]. High intertidal salt flats, or sabkha, are being identified as important blue carbon ecosystems in arid regions (e.g. [17–19]). Other ecosystems that have been identified as potential blue carbon ecosystems include low intertidal mud flats, marine sediments, coralline algae (maerl and rhodolith beds) and coral reef lagoons [2]. For ecosystems such as rocky reefs, polar zoobenthos and shellfish reefs, the evidence remains patchy or inconclusive for one or more of the criteria [1,2].

Although all potential ecosystems may not meet all criteria, the high connectivity among coastal and marine ecosystems, for example, through biogeochemical fluxes and habitats for fauna, have led to suggestions that landscape-scale and perhaps ocean basin-scale approaches to blue carbon are needed [20,21]. The urgent need to achieve climate mitigation and the KMGBF and Sustainable Development Goals may further stimulate science to incorporate blue carbon and adjacent ecosystems at scales beyond the relatively small scale of blue carbon projects that are mostly focused on single ecosystems. In many cases, the most pressing opportunity for gathering attention and finance for conservation and restoration may not be for carbon, but instead for the conservation of biodiversity and the ecosystem service benefits derived from connected ecosystems within the broader landscape.

In addition to scientific insights, technological innovations in mapping and monitoring blue carbon ecosystems have rapidly developed since 2019. Global maps of ecosystem extent from satellite remote sensing combined with machine learning models are available for mangroves, tidal marshes, coral reefs (and lagoons), kelp, seaweed aquaculture and low intertidal mud flats (e.g. [22–26]) with seagrass maps not far off, despite the difficulties of detecting seagrass underwater [27]. Assessment methods using unmanned autonomous vehicles (UAV) or drones are also increasingly available (e.g. [28]). Maps of synthesized soil carbon data (often done using data fusion and machine learning models) are available for mangroves [29], tidal marshes [30] and seagrass [31] and aboveground biomass maps of mangroves have been refined globally [32]. These technological innovations enhance the capacity for verification of conservation and restoration actions at broad scales and at high resolutions, thereby increasing confidence in detecting and estimating carbon and other benefits from management of blue carbon ecosystems.

## 3. Blue natural capital as an investable asset

The language of finance has always been a part of the blue carbon dialogue because the original intention was to increase attention to and investment in conservation and restoration of coastal and marine ecosystems for the benefit of climate, biodiversity and ultimately people [5,33]. While the science has matured, so too has knowledge of how to finance conservation and restoration of blue carbon ecosystems. A focus on carbon markets and emerging biodiversity certifications is being augmented by conceptualizing blue carbon ecosystems as investable natural assets.

The voluntary carbon markets have held a strong interest for blue carbon [7,34], and although expanding in scope and size, the development of blue carbon markets has been slow for a range of reasons associated with social, financial, biophysical and technological factors [34,35]. Social barriers have included inadequate policy development to support blue carbon markets, unclear governance of blue carbon resources, variable land-tenure arrangements, regulatory impediments, uncertainty in carbon rights, limited engagement and benefit sharing with local resource owners and users, lack of social licence, limited investment models and methods and high costs [34,36]. The expansion of blue carbon markets has also been hampered by limited supply of projects that may in part be associated with the naturally small scale of coastal ecosystems in many locations, as well as limited knowledge of restoration methods and opportunities [35,37].

The barriers to increasing the number of blue carbon projects have stimulated actions to overcome them, for example, by introducing new accounting methods and improving earlier ones over a range of platforms [14,17,34], identifying where conservation and restoration could take place [37–39], reducing costs of verification of blue carbon projects by using remote sensing (e.g. [40]), and in advancing best practices in project development for ecosystem restoration and nature-based solutions [41–43] (e.g. codes of practice for achieving high-quality projects [44]). With emerging biodiversity certification methods (e.g. VERRA Climate, community and biodiversity standards [45]), stacking payments for carbon and biodiversity (and other ecosystem services) could increase the opportunities for developing projects and the feasibility of blue carbon conservation and restoration projects, given the high costs of implementation. Biodiversity markets, however, come with similar risks to those for carbon markets, including standards that enable biodiversity losses (e.g. if offsets are allowed) [46] and by reinforcing existing inequalities among the global north and south [47].

With the background of the progress made so far, how can we accelerate investment in restoration and conservation of blue carbon ecosystems? The financial and insurance sectors have offered possibilities, some of which require thinking big, to match the size of blue carbon activities with the size of large financial instruments [4]. Some of these financial instruments allow blue carbon stewardship payments that are not possible when using market-based methodologies because ongoing stewardship is not considered ‘additional’ to business-as-usual in carbon markets [21]. However, when using large financial instruments, management of blue carbon ecosystems for climate mitigation may be just one component of a larger program of work.

Investments in large blue natural capital projects can be supported by governments through redirection of harmful subsidies (e.g. for unsustainable commodities, excessive fertilizer-use and land clearing), alignment of incentives (e.g. national regulations and policies for carbon and biodiversity and taxation) and insurance that reduces risks to investors [4]. Despite the identification of a wide range of finance types that can be used to finance large natural capital projects including loans, bonds and debt swaps, blended finance (finance from governments and philanthropic funds used to mobilize private sector capital by derisking private capital), impact investments (e.g. Bezos Earth Fund) and equities, global investment remains limited [4].

Examples of large-scale blue natural capital projects include a debt swap and the issuance of a blue bond for the Seychelles in 2018, which was used to create a marine spatial plan and the Seychelles Conservation and Climate Adaptation Trust (SeyCCAT) that annually funds projects to maintain, restore and educate on marine ecosystems (similar arrangements have been implemented in Belize, Barbados and the Bahamas; [48] or the Small-Scale Fisheries Impact Bond by RARE for Southeast Sulawesi, Indonesia that supports fisheries management and the creation of marine reserves [49]. The bond outcome funders (a mix of government, NGO and philanthropists) are committed to repaying investors based on verified outcomes. Bonds can be bought and sold (they are liquid) which is attractive for many investors. Additional models for financing of conservation and restoration of blue carbon ecosystems may come from those used to enhance human health. For example, the ridge-to-reef management of water resources in the Pacific is an example of an approach where management of blue carbon ecosystems could be included (along with other ecosystems) at the seascape level, not only for their role in supporting human health [50] but also for climate mitigation services, effectively enhancing the value of projects. Achieving these holistic, integrated projects requires collaborative transdisciplinary approaches (and people trained in these approaches) and coordination.

The risks of these large-scale financial arrangements include complexities in allocating the raised funds because they require high levels of coordination and secure governance to demonstrate the impact of the funding (e.g. for biophysical and social outcomes, new guidelines are emerging, ADB 2023) [51]. So far, USD $5 billion has been invested in 26 blue bonds [52] (far short of the USD$520 billion estimated that will be needed by 2030 [4]).

Framing blue carbon ecosystems as blue natural capital could be an important step in increasing investment. Investing in natural assets is increasingly recognized as an attractive business case underpinning the economic performance of nations, which accrue annual gains of USD$100–350 billion, with the largest percentage gains in the lowest income countries [53]. However, grasping this demonstrated potential requires widespread development and uptake of appropriate financial instruments and social safeguards.

## 4. Research to support large-scale conservation and restoration of blue carbon ecosystems

Co-benefits have always been an important part of the blue carbon narrative, and these benefits are being increasingly well characterized to guide investment (e.g. [54,55]). Characterizing biodiversity in blue carbon ecosystems, the dependence of flora and fauna on connected ecosystems at landscape scales, and building knowledge of the links between biodiversity and ecosystem services are important research topics that will underpin carbon and biodiversity markets and finance for blue natural capital projects. Future research can strive to redress imbalances in data availability for carbon and biodiversity (and other ecosystem services) among the global north and south to support fair carbon and biodiversity standards [56]. Research to develop and evaluate robust, cost-effective, community-led monitoring for verification of carbon and biodiversity outcomes would empower communities, increase confidence in the outcomes of projects and incentivize investment.

The Intergovernmental Panel on Climate Change (IPCC) guidelines for Greenhouse Gas (GHG) Inventories underlie many voluntary carbon market methods, but they currently lag behind the global data available in blue carbon research. The lag has negative impacts for implementing carbon projects at all scales. This is because projects that use IPCC default values for carbon stocks and fluxes (usually those from the global south where there is often limited research support) are penalized for high levels of uncertainty by existing verification protocols and therefore receive fewer carbon credits, and because there is limited guidance on the range of activities, ecosystems, carbon pools and fluxes that could be in scope [21]. Updates to IPCC guidance are requested by Parties to the United Nations Framework Convention on Climate Change that then issue a mandate to the IPCC. Updating IPCC guidance for additional ecosystems (figure 1), overlooked geographies and activities (e.g. restoration) are key to enabling accurate, trusted and globally agreed on parameters for blue carbon assessments.

In addition to standards and monitoring for carbon and biodiversity, the development of standardized metrics for evaluating climate change adaptation and other ecosystem services (e.g. water quality, fisheries and cultural services) provided by blue carbon ecosystems is important for characterizing benefits from investments. Monitoring changes in levels of threats will also be useful (e.g. [57]). The System of Environmental Economic Accounts (UN SEEA), enabled by new technology and adapted for blue carbon (e.g. [58–60]) can help characterize the benefits of managing blue carbon ecosystems at large scales needed for investments in blue carbon ecosystems and for assessment of returns on investments in blue carbon natural capital. Broad-scale implementation of UN SEEA requires, however, capacity building, as less than 30 nations were able to implement the system due to multiple limitations (e.g. difficulties in defining ecosystem condition indicators and coordinating data synthesis from multiple institutions) [61].

Investors are sensitive to risks, and while many risks can be managed, climate change is increasing risks to coastal ecosystems and blue carbon. Risks from climate change include those of sea level rise and extreme events (storms, droughts, changes in groundwater, flooding and thermal events), with threats often interacting with anthropogenic stressors in synergistic ways [57,62]. Currently, there is global under-investment in characterizing and responding to climate risks [63]. Research at local and global scales is needed to devise climate-smart approaches to conservation and restoration of blue carbon and blue natural capital (e.g. in marine spatial planning), including the development of multi-ecosystem, landscape models (e.g. [57,64]) and accessible tools linked to downscaled climate predictions [65]. The insurance industry could be actors for good, helping to drive climate-smart projects that reduce risks (e.g. parametric insurance), as well as directly investing in blue natural capital [4].

There is limited research on the governance of blue carbon ecosystems or blue carbon projects, yet governance systems provide the safeguards to avoid environmental damage and inequalities [66–68]. Complexity in small-sized blue carbon projects already poses governance challenges [66]. Devising fair and equitable governance systems for blue natural capital projects is challenging given the potential large scale, multiple communities, stakeholders and contexts over which blue natural capital projects could occur [68]. We seem surprisingly unprepared for the governance of large projects, even in countries with strong governance models, and given the large-scale interventions nations are committed to under the Paris Agreement and the KMGBF [69].

**Figure 2.**
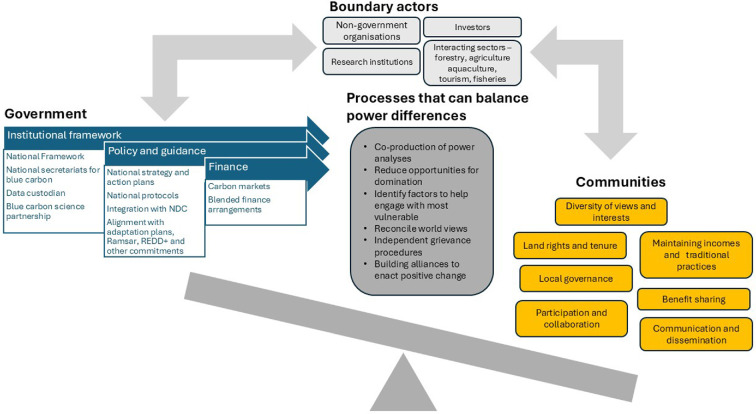
In implementing blue carbon strategies, power differentials occur between government and its planning and implementation processes (in blue, adapted from [70]) and communities and their interests (orange, adapted from [68]). Boundary actors (light grey) work with both communities and government [71]. There are a range of processes that can help to redress power imbalances between government and communities in dark grey (adapted from [68]). Boundary actors can influence power and have an important role to increase the power of communities within governance systems.

Studies of governance for blue carbon in Indonesia provide an example of the complexities faced by governments in developing frameworks and policy, implementing laws and policies and coordinating the diverse range of institutions at all levels of government that are needed to support blue carbon activities [70]. Endorsements from the highest level (e.g. in Indonesia from the President) and incentives for aligning the activities of different government agencies appear fundamental. Experience in governance of blue carbon projects implemented in Kenya, Madagascar and Colombia (and other nations) has provided a detailed understanding of how governance could be improved and how power differentials can be managed (figure 2). Governance can be improved through processes that reduce power imbalances. A number of mechanisms have been proposed [68] to help reduce power imbalances including: co-investigation of power among government, communities and others of where power is held; reducing opportunities for domination by particular institutions and actors; identifying how to engage with the most vulnerable within communities; reconciling differences in viewpoints held by different actors; establishing independent and transparent processes to address grievances and building alliances to act for change, for example, through establishment of umbrella organizations that mitigate risks of conflict among stakeholders and time-inconsistencies in the delivery of projects. Actors and institutions that span the boundary between government and communities (e.g. boundary actors, including non-government organizations, researchers and their organizations, investors and actors from industries) can have an important role in enhancing power held within communities by supporting processes that are known to reduce power imbalances [71], and thus can contribute to strengthening the position of communities within governance systems.

Polycentric governance systems, where multiple interacting sub-systems, including local community organizations, coordinate within the governance system, are proposed to be superior arrangements for natural resource management as they are flexible, adaptive and inclusive [72]. But polycentric governance for coastal ecosystems and blue carbon has rarely been assessed. An investigation of the governance of adaptation to sea level rise in California found evidence of increasing polycentric governance over time (decades) because of the rising importance of local stakeholder organizations and collective actions [73]. Although the effectiveness of different governance structures for adaptation to sea level rise was not evaluated, this study suggested the polycentric governance structure gave rise to shared learnings among actors, integration of different institutions managing different components of the landscape (e.g. those managing water, vegetation, infrastructure) at local scales (the scale at which the impacts of sea level rise are felt), and strong coordinating responsibilities for regional organizations [73]. For blue natural capital projects, the takeaway lesson may be that powerful local institutions in conjunction with regional coordinating institutions are an effective arrangement for management of blue carbon ecosystems, although the effectiveness of governance arrangements is often context-dependent [67,73]. There is a strong need for research devising and evaluating governance systems that can deliver effective blue carbon and blue natural capital projects in ways that are just and equitable.

## 5. Conclusions

Global attention to blue carbon has been effective in stimulating conservation and restoration activities in mangrove, seagrass and tidal marsh ecosystems with expanding science in other ecosystems. Science has expanded exponentially in the last decade, and impacts can be seen in the Nationally Determined Contributions and many other government policy and industry documents, and their investments. While the scientific agenda on blue carbon continues to be populated with new and important questions, the challenge to deliver on the promise of blue carbon seems to rest less on scientific uncertainties, but instead on interacting elements of communities, finance and governance of blue carbon social–ecological systems. Blue carbon markets offer only a portion of the finance that is needed to achieve targets for both the Paris Agreement and the KMGBF. We need to think outside the narrow blue carbon market box to develop additional financial instruments and enhance governance systems that will allow capture of the full scale of finance possibilities available for conservation and restoration of blue carbon ecosystems. This requires large-scale initiatives which could include blue natural capital projects spanning landscapes of connected ecosystems (up to regional scales) and that are, therefore, embedded in complex social–ecological systems. Building capacity in trans-disciplinary scientists will be key to success. There are risks following this pathway; some of the largest are those associated with the adequacy of governance systems to prevent harm. The dimensions and actors involved in blue carbon continue to expand, and perhaps this can be used to harness the growing opportunities for novel financing of conservation and restoration underpinned by science and robust governance.

## Data Availability

This article has no additional data.
